# International Myeloma Working Group risk stratification model for smoldering multiple myeloma (SMM)

**DOI:** 10.1038/s41408-020-00366-3

**Published:** 2020-10-16

**Authors:** María-Victoria Mateos, Shaji Kumar, Meletios A. Dimopoulos, Verónica González-Calle, Efstathios Kastritis, Roman Hajek, Carlos Fernández De Larrea, Gareth J. Morgan, Giampaolo Merlini, Hartmut Goldschmidt, Catarina Geraldes, Alessandro Gozzetti, Charalampia Kyriakou, Laurent Garderet, Markus Hansson, Elena Zamagni, Dorotea Fantl, Xavier Leleu, Byung-Su Kim, Graça Esteves, Heinz Ludwig, Saad Usmani, Chang-Ki Min, Ming Qi, Jon Ukropec, Brendan M. Weiss, S. Vincent Rajkumar, Brian G. M. Durie, Jesús San-Miguel

**Affiliations:** 1 Servicio de Hematologia, Hospital Universitario de Salamanca, Centro de Investigación del Cáncer, Instituto de Biología Molecular y Cellular del Cáncer (Universitario de Salamanca-Consejo Superior de Investigaciones Científicas), Salamanca, Spain; 2 Division of Hematology, Mayo Clinic, Rochester, MN USA; 3 Hematology and Medical Oncology, Department of Clinical Therapeutics, National and Kapodistrian University of Athens, School of Medicine, Athens, Greece; 4 Department of HematoOncology, University Hospital Ostrava and Faculty of Medicine, Ostrava University, Ostrava, Czech Republic; 5 Department of Hematology, Amyloidosis and Myeloma Unit, Hospital Clínic, IDIBAPS, University of Barcelona, Barcelona, Spain; 6 Perlmutter Cancer Center, NY Langone Health, New York, NY USA; 7 Department of Molecular Medicine, Amyloidosis Research and Treatment Center, Foundation ‘Istituto di Ricovero e Cura a Carattere Scientifico (IRCCS) Policlinico San Matteo’, University of Pavia, Pavia, Italy; 8 Department of Internal Medicine V, University Medical Hospital and National Center of Tumor Diseases, Heidelberg, Germany; 9 Department of Clinical Hematology, Centro Hospitalar e Universitário de Coimbra, Coimbra, Portugal; 10 Department of Medicine, Surgery and Neurosciences, University of Siena, Siena, Italy; 11 Department of Haematology, London North West Healthcare, London, UK; 12 Service d’Hématologie, Hôpital Pitié Salpêtrière, Paris, France; 13 Department of Hematology, Sahlgrenska University Hospital, Goteborg and Skane University Hospital, Lund, Sweden; 14 Department of Experimental, Diagnostic and Specialty Medicine – DIMES, University of Bologna, Bologna, Italy; 15 Department of Hematology, Hospital Italiano de Buenos Aires, Buenos Aires, Argentina; 16 Department of Hematology, Hopital de La Milétrie, CHU, Poitiers, France; 17 Department of Hemato-Oncology, Hallym University Dongtan Sacred Heart Hospital, Hwasung, South Korea; 18 Department of Hematology, Hospital de Santa Maria, Lisboa, Portugal; 19 Department of Medicine, Center for Oncology, Hematology and Palliative Care, Wilhelminenspital, Vienna, Austria; 20 Department of Hematology and Medical Oncology, Atrium Health/Levine Cancer Institute, Charlotte, NC USA; 21 Hematology, Department of Internal Medicine, Seoul St. Mary’s Hospital, The Catholic University of Korea, Seoul, South Korea; 22 Janssen Pharmaceuticals, Horsham, PA USA; 23 Cedars-Sinai Outpatient Cancer Center, Los Angeles, CA USA; 24 Clinica Universidad de Navarra, CIMA, CIBERONC, IDISNA, Pamplona, Spain

**Keywords:** Myeloma, Cancer epidemiology

## Abstract

Smoldering multiple myeloma (SMM) is an asymptomatic precursor state of multiple myeloma (MM). Recently, MM was redefined to include biomarkers predicting a high risk of progression from SMM, thus necessitating a redefinition of SMM and its risk stratification. We assembled a large cohort of SMM patients meeting the revised IMWG criteria to develop a new risk stratification system. We included 1996 patients, and using stepwise selection and multivariable analysis, we identified three independent factors predicting progression risk at 2 years: serum M-protein >2 g/dL (HR: 2.1), involved to uninvolved free light-chain ratio >20 (HR: 2.7), and marrow plasma cell infiltration >20% (HR: 2.4). This translates into 3 categories with increasing 2-year progression risk: 6% for low risk (38%; no risk factors, HR: 1); 18% for intermediate risk (33%; 1 factor; HR: 3.0), and 44% for high risk (29%; 2–3 factors). Addition of cytogenetic abnormalities (t(4;14), t(14;16), +1q, and/or del13q) allowed separation into 4 groups (low risk with 0, low intermediate risk with 1, intermediate risk with 2, and high risk with ≥3 risk factors) with 6, 23, 46, and 63% risk of progression in 2 years, respectively. The 2/20/20 risk stratification model can be easily implemented to identify high-risk SMM for clinical research and routine practice and will be widely applicable.

## Introduction

Smoldering multiple myeloma (SMM) represents a transitional stage between monoclonal gammopathy of undetermined significance (MGUS) and active multiple myeloma (MM)^1,2^. Before International Myeloma Working Group (IMWG) revised the MM diagnostic criteria, SMM was defined by the presence of either a serum monoclonal protein of ≥3 g/dL (or ≥500 mg/24 h in urine or both) and/or ≥10% bone marrow plasma cells (BMPCs) without evidence of any CRAB symptoms (hypercalcemia, renal impairment, anemia, or lytic bone lesions). SMM is a heterogeneous disease with a risk of progression to MM of 10% per year during the first 5 years following diagnosis, decreasing to 3% per year over the subsequent 5 years, and 1% per year after the 10 years of diagnosis^3^. The Mayo Clinic and the Spanish Group had previously created different risk stratification models that can identify SMM patients with a 2-year risk of progression to MM of ≥50%. The Mayo Clinic model included M-protein (≥3 g/dL), BMPCs infiltration (≥10%), and the ratio of serum free light chain (sFLC) (≥8 or <0.125) to categorize patients into three subgroups, with a 52% risk of progression in 2 years for those presenting with all three risk factors^4^. The Spanish model used the presence of ≥95% clonal plasma cells among all BMPCs by immunophenotyping and the presence of immune paresis and identifies a subgroup of patients having both features with a 50% risk of progression at 2 years^5^.

The standard of care for SMM, irrespective of the risk status of the patients, has been observation. However, there are two randomized studies confirming the benefit of early treatment in reducing the risk of progression to MM. The Spanish Myeloma Group conducted the first phase III trial in high-risk SMM, comparing lenalidomide plus dexamethasone (Ld) versus observation, and Ld significantly delayed the progression to MM and patients lived longer (overall survival (OS))^6^. The Eastern Cooperative Oncology Group (ECOG) group has also reported a significant benefit for the early treatment with single-agent lenalidomide versus observation in SMM^7^.

Although the Spanish trial did not lead to a change in the standard of care, it encouraged the IMWG to revise the criteria for the definition of SMM and MM to identify a subset of SMM with a 2-year risk of progression of approximately 80% who were then categorized as MM. The identification of this subgroup is based on the presence of any one of these biomarkers: BMPCs ≥60% or involved/uninvolved sFLC ratio ≥100, and presence of >1 focal lesion on magnetic resonance imaging, in patients having bone marrow with at least 10% of clonal BMPC infiltration. This led to a paradigm shift as these patients were then regarded as having myeloma and were offered therapy^8^.

Other groups, in parallel, also created different models to identify SMM patients with a progression risk at 2 years of 50% using different features like positive uptake on positron emission tomography–computed tomography (PET-CT), type of M-protein, cytogenetic abnormalities, increase in serum M-protein and decrease in hemoglobin count over time, presence of Bence Jones proteinuria, or genetic signatures, among others^9–14^. At the same time, many clinical trials are ongoing in high-risk SMM according to different risk models, and all these developments brought to the fore two issues: (i) the heterogeneity of the different risk models developed using rather small series of SMM patients and (ii) the real impact of the revised SMM definition. This forms the basis of the current study.

We studied a large cohort of SMM patients to identify factors that predicted progression to MM with the goal of developing an easy risk score to predict the 2-year progression risk. The factors identified were M-protein (>2 g/dL), BMPCs infiltration (>20%), and the ratio of involved versus uninvolved sFLC (>20). In addition, we have also created a risk score that accounts for the entire range of the variable measurements, which can be used to provide a more individualized 2-year risk of progression for every SMM patient.

## Patients and methods

Patients with SMM diagnosed after January 2004 were included in this retrospective medical chart review from participating IMWG sites globally. The SMM definition required to be included in the study was based on the 2014 IMWG criteria^8^. Patients included in this analysis required baseline data from diagnosis (+/−3 months), no progression to MM or other plasma cell disorders within 6 months from diagnosis, a minimum follow-up of 1 year, and should not have been included in any therapeutic trial.

Trained staff collected data from the patient medical charts in three case report forms: (i) patient registration and demographics form, (ii) diagnosis and baseline assessment form, and (iii) 12-month assessment form. The Institutional Review Board at each site approved the study. The study was conducted in accordance with the Declaration of Helsinki and the International Council for Harmonisation Guidelines.

The primary objective was to develop a risk stratification model that will identify SMM patients who have high risk of progression to MM or other plasma cell disorders (50% progression risk within the first 2 years from diagnosis) based on the 2014 IMWG criteria for definition of both SMM and MM.

Time to progression (TTP) to MM or amyloidosis was the primary end point and was defined as the time elapsed between diagnosis of SMM and when the patient experienced progressive disease. All patients who did not progress at the time of last follow-up were censored in the TTP analysis. A secondary objective was to create a risk scoring tool that would allow individualized estimate of risk at various time points from diagnosis.

### Statistical methods

Due to the nature of the study, no formal hypothesis was used to calculate the sample size. We summarized categorical variables as proportions and continuous variables as medians (range). Univariate Cox regressions were run for each factor to assess an initial relationship between a factor and progression to MM or related disorders. For each factor where *p* ≤ 0.25, optimal cut points were identified using Youden’s Index. Receiver operating characteristic (ROC) curves were generated, with the cut-off maximizing both sensitivity and specificity determined. Sensitivity and specificity were reported for these optimal cut points. Interaction term between candidate variables were included in the model selection if candidate variables with correlations satisfy the above criteria. Using the optimal cut points, a stepwise regression analysis was used to determine predictive variables using a required selection stay criterion of 0.001. A random forest algorithm consisting of an ensemble of decisions trees^15^ created using the random Forest package was used to confirm variable selection^16^. Important prognostic variables for predicting probability of progression at 2 years were identified and ranked based on the percentage change in mean squared error. Similar methods were used to identify the most significant cytogenetic abnormalities associated with progression to MM in patients with available information.

Patients were categorized based on the number of risk categories present and Cox proportional hazard regression models were used to calculate hazard ratios (HRs) and 95% confidence intervals (CIs). Kaplan–Meier curves were used to illustrate TTP across the number of risk categories present.

A risk score was developed to elucidate the potential strength of individual relationships between each of the previously defined variables based on the method described by Sullivan et al.^17^. Variables were categorized based on clinically relevant as well as spline functions to establish the general trends in the risk of progression with increasing laboratory values. Scores for each risk factor were assigned relative weights of each coefficient in the multivariable regression model. Predicted risk of progression were summarized based on this scoring tool. Analyses were conducted in SAS version 9.4 (The SAS Institute, Cary, NC) and R Core Team (2018).

## Results

### Patient characteristics

One thousand nine hundred and ninety-six SMM patients from 75 centers in 23 countries were included. Baseline characteristics are in Table 1. The median age at diagnosis was 64 years (interquartile range (IQR): 56–72). The median serum M-component and BMPC infiltration were 1.8 g/dL (IQR: 1.1–2.6) and 15% (IQR: 12–25), respectively. Concerning cytogenetic information (available in 689 patients), trisomies and del13q/monosomy 13 were the most frequent abnormalities (31.4% and 28.4%, respectively). 1q gain was reported in 25.4% and t(4;14) and del(17p/monosomy 17) in 10.2% and 5.4%, respectively.

**Table 1:** Baseline characteristics of the study population.

	Missing data (*n*)	Quantity, median (IQR) or %
Age (years), median (range)	0	64 (56–72)
Gender (Male), %	0	978 (49%)
Hemoglobin (g/dL)	54	12.9 (11.8–13.8)
Creatinine (mg/dL)	136	0.88 (0.71–1.04)
Calcium (mg/dL)	172	9.3 (9–9.7)
Albumin (g/dL)	179	4 (3.7–4.3)
Beta-2 microglobulin (mg/dL)	357	2.3 (1.8–3)
Serum M-protein (g/dL)	0	1.8 (1.1–2.6)
Serum FLC ratio	633	6.2 (2.1–24.1)
Heavy-chain type	120	
IgA		452 (24.1%)
IgD		6 (0.3%)
IgG		1402 (74.7%)
IgM		16 (0.9%)
Light-chain type	24	
Kappa		1199 (60.8%)
Lambda		773 (39.2%)
Immunoparesis^a^	235	992 (56.3%)
Urine M-spike (mg/24 h)	792	0 (0–30)
BMPC^b^, higher of biopsy and aspirate %	0	15 (12–25)

^a^Immunoparesis was defined as reduction in one or more of the uninvolved immunoglobulin level below normal range.

^b^Bone marrow plasma cell percentage.

### Survival outcomes

**Fig. 1:**
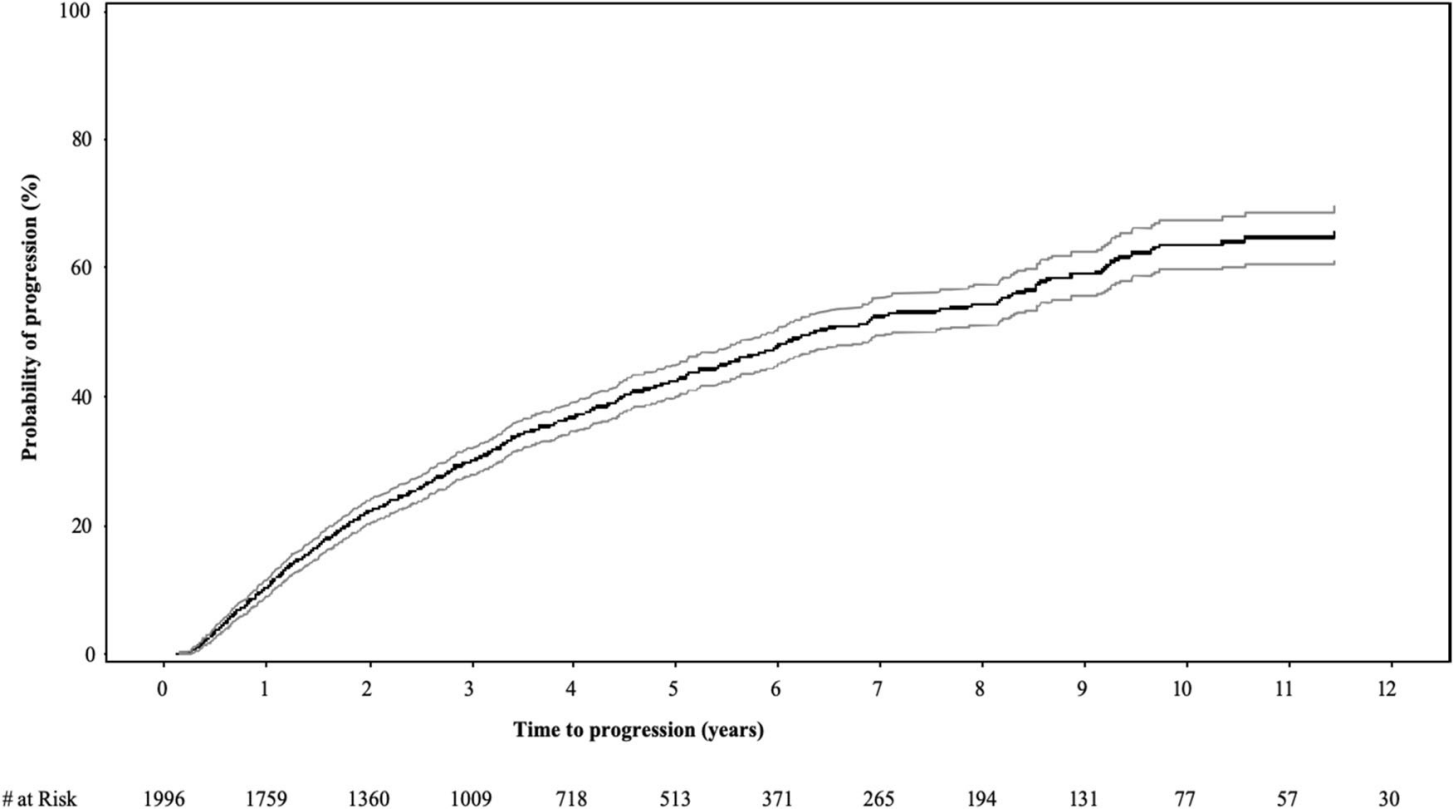
Probability of progression over time in the full study cohort (*n* = 1996). The median time to progression for the entire cohort was 6.4 years (95% CI 6.0–7.2); the 2-, 5-, and 10-year risk of progression were 22, 42, and 64%, respectively.

The median follow-up from diagnosis was 3.0 years (IQR 1.6–5.1). At the data cut-off, 815 (41%) patients had progressed to MM or a related disorder. The median TTP for the entire cohort was 6.4 years (95% CI 6.0–7.2); the 2-, 5-, and 10-year risk of progression were 22, 42, and 64%, respectively (Fig. 1). The estimated 5- and 10-year OS for the entire cohort from SMM diagnosis was 93.8% (92.0–95.2) and 88.3% (84.8–91.1), respectively.

### Risk factors for progression and stratification model

The factors initially included in the univariate analysis for predicting progression risk at 2 years from the diagnosis are shown in Table 2 indicating those included in the model because of their capacity for predicting risk of progression to MM at 2 years (*p* value ≤ 0.25).

**Table 2:** Identification of factors for risk stratification model.

	*p* value	Candidate factors (*p* value <0.25)	Hazard ratio (95% CI)
Age (per 10 years)	0.034	Yes	1.09 (1.01, 1.19)
Female sex	0.4552	No	0.93 (0.77, 1.13)
Hemoglobin (g/dL)	<0.0001	Yes	0.87 (0.82, 0.93)
Creatinine (mg/dL)	0.1192	Yes	1.2 (0.96, 1.5)
Calcium (mg/dL)	0.6844	No	0.98 (0.9, 1.07)
Albumin (g/dL)	0.0786	Yes	0.84 (0.68, 1.02)
Serum M-protein (g/dL)	<0.0001	Yes	1.09 (1.06, 1.12)
Beta-2 microglobulin (mg/dL)	<0.0001	Yes	1.21 (1.14, 1.29)
Absolute difference Kappa–Lambda (mg/dL), per 100	<0.0001	Yes	1.03 (1.01, 1.04)
Involved to uninvolved sFLC ratio, per 100	<0.0001	Yes	1.1 (1.06, 1.13)
Heavy-chain type (IgG versus IgM)	0.5003	No	0.92 (0.73, 1.17)
Heavy-chain type (IgG versus IgA)	0.3336	No	0.92 (0.78, 1.09)
Light-chain type (Kappa versus Lambda)	0.0611	Yes	0.83 (0.68, 1.01)
Immunofixation	0.9641	No	n/a
Immunoparesis^a^	<0.0001	Yes	1.53 (1.24, 1.89)
Urine M-spike (mg/24 h), per 1000	0.163	Yes	1.05 (0.98, 1.14)
BMPC^b^ %, per 10	<0.0001	Yes	1.44 (1.36, 1.52)

*n/a* not applicable.

^a^Immunoparesis was defined as reduction in one or more of the uninvolved immunoglobulin level below normal range.

^b^Bone marrow plasma cell percentage

From among these, the stepwise model selection and random forest algorithm identified serum M-protein concentration, involved to uninvolved sFLC ratio, and BMPC percentage as the most relevant factors predicting progression to MM, and based on Youden’s Index in the ROC analyses, the optimal cut-offs for the risk factors were 1.9 g/dL for serum M-protein (specificity 60% and sensitivity 71%), 19.3 for the involved to uninvolved sFLC ratio (specificity 79% and sensitivity 55%), and 16.4% for the BMPC infiltration (specificity 55% and sensitivity 75%) as shown in Table 3. For convenience and simplicity, we decided to use 2 g/dL, 20%, and 20% as cut-off levels for these factors. Using these cut points, multivariable analysis identified the presence of serum M-protein >2 g/dL as an independent prognostic factor predicting high risk of progression to MM (HR: 2.07, 95% CI: 1.62–2.65), together with involved to uninvolved sFLC ratio >20 (HR: 2.66, 95% CI: 2.09–3.38) and BMPC infiltration >20% (HR: 2.39, 95% CI: 1.87–3.05).

**Table 3:** Receiver operating characteristic (ROC) curves with area under the curve (AUC) analysis to identify optimal cut-offs for continuous risk factors (identified from Table 2) with respect to progression to MM within 2 years.

	Cut point	Specificity (%)	Sensitivity (%)
Age (per 10 years)	57	28	80.6
Hemoglobin (g/dL)	13.3	39.9	73.3
Creatinine (mg/dL)	0.81	42.9	66
Albumin (g/dL)	4.09	46.4	64
Serum M-protein (g/dL)	1.91	60	70.6
Beta-2 microglobulin (mg/dL)	2.57	65.7	52.8
Absolute difference Kappa-Lambda (mg/dL), per 100	18.5	66	54.6
Involved to uninvolved sFLC ratio, per 100	19.3	78.7	54.7
Urine M-spike (mg/24 h), per 1000	75	83.1	29.8
BMPC, higher of biopsy and aspirate %	16.4	58.1	71.8

**Fig. 2:**
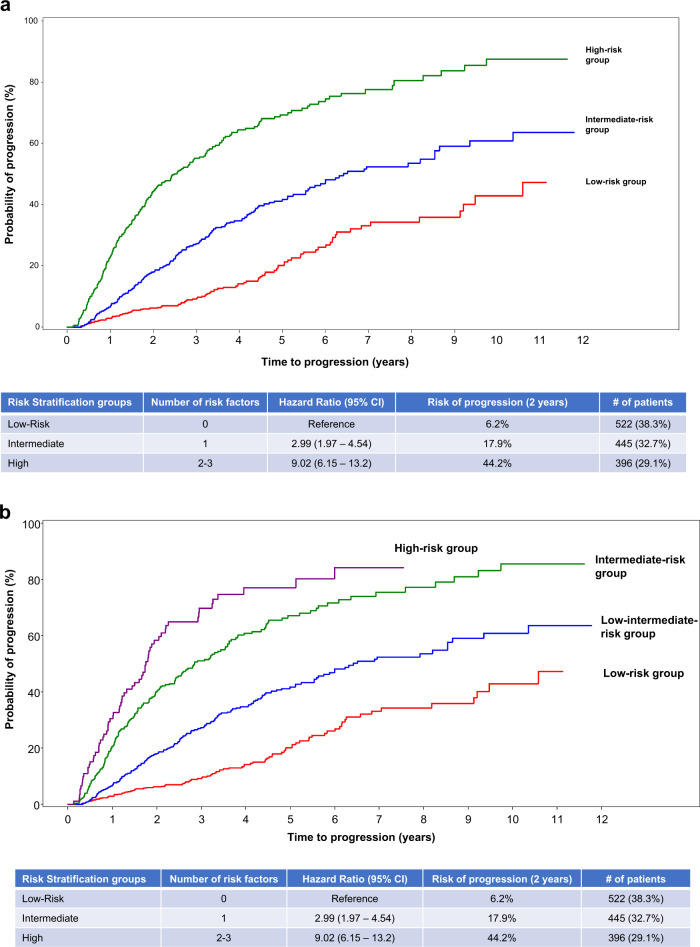
Risk of progression at 2 years based on presence or absence of risk factors in patients with smoldering multiple myeloma. **a** Probability of progression at 2 years in the three different subgroups of patients according to the model 2/20/20. Patients with no risk factors (low-risk group) had a risk of progression at 2 years of 6%, those with one factor (intermediate-risk group) had a risk of progression of 18% at 2 years, and those with ≥2 factors (high-risk group) had a 44% progression risk at 2 years. **b** Probability of progression at 2 years according to the model 2/20/20 with separation of high-risk group based on presence of 2 or 3 risk factors. Of the 396 patients included in the high-risk group, 92 presented with the three risk factors and had a slightly higher risk of progression to MM.

We then proceeded to create a risk stratification model including 1363 patients in whom all three factors were available: five hundred and twenty-two patients (38%) did not present any of the factors (reference group) with a risk of progression at 2 years of 6% (the low-risk group); 1 out of the 3 factors was identified in 445 patients (33%) with a 18% risk of progression to MM at 2 years (HR: 2.99, 95% CI: 1.97–4.54) (the intermediate-risk group); and the high-risk group defined by the presence of 2 or 3 risk factors included 396 patients (29%) with a 44% progression risk at 2 years (HR: 9.02, 95% CI: 6.15–13.2) (Fig. 2a). Of the 396 patients included in the high-risk group, 92 presented with the 3 risk factors and had a slightly higher risk of progression to MM (Fig. 2b).

**Fig. 3:**
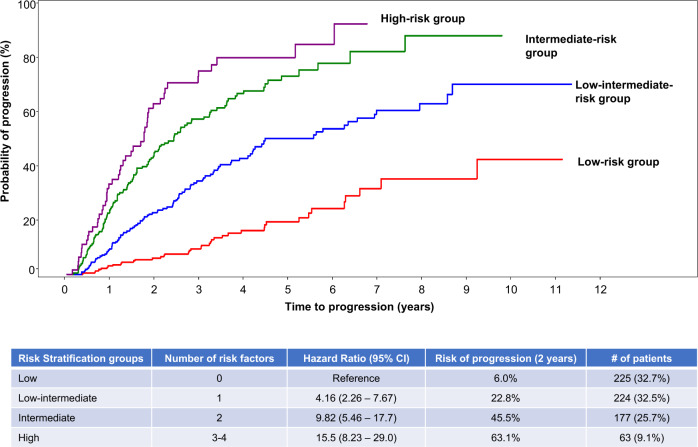
Probability of progression at 2 years in the four different subgroups of patients according to the model 2/20/20 plus cytogenetic abnormalities (t(4;14), t(14;16), +1q, and/or del13q/monosomy 13). This model defined four groups of SMM patients: low risk with none of the factors had a progression risk at 2 years of 6%, low–intermediate with one factor present had a progression risk at 2 years of 23%, intermediate risk with the presence of 2 factors had a risk of progression at 2 years of 37%, and the high risk with ≥3 of the factors had a progression risk at 2 years of 63%.

We explored the added value of the presence of any cytogenetic abnormalities to the 2/20/20 risk model if all factors were available (689 patients). The stepwise model selection identified the presence of t(4;14), t(14;16), +1q, and del13q/monosomy 13 by fluorescence in situ hybridization (FISH) as the most relevant ones. This model defined four groups of SMM patients with different progression risk at 2 years (Fig. 3): low risk with a progression risk at 2 years of 6% (*n* = 225; 33%) and defined by the presence of none of the factors; low–intermediate (*n* = 224; 33%) if one factor was present and the progression risk at 2 years was 23% (HR: 4.16, 95% CI: 2.26–7.67); intermediate risk (*n* = 177; 26%) defined by the presence of 2 factors and risk of progression at 2 years of 46% (HR: 9.82, 95% CI: 5.46–17.7); and the high risk (*n* = 63; 9%) with a progression risk at 2 years of 63% (HR: 15.5, 95% CI: 8.23–29.0) if ≥3 of the factors were present.

### Scoring tool to predict risk of progression at 2 years

**Fig. 4:**
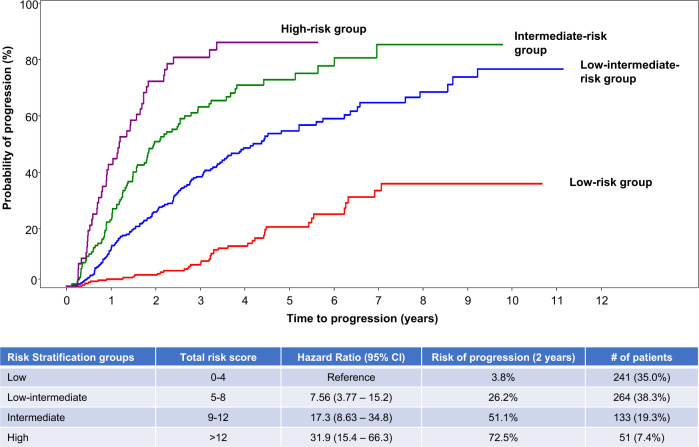
Risk of progression according to the risk score. Risk score was developed using the entire range of the values for BMPC, serum FLC, and serum M-spike as well as cytogenetic abnormality. Patients with total risk score between 0 and 4 had a 2-year progression risk of 3.8%, patients with a total score between 5 and 8 had a risk of 26%, those with a score between 9 and 12 had a risk of progression of 51%, and those with a score >12 had a risk of progression of 73%.

Based on the univariate and multivariate analysis previously conducted, the three risk factors (serum M-protein, involved to uninvolved sFLC ratio, and BMPC infiltration) together with the cytogenetic abnormalities were included in a logistic regression model for creating a scoring tool to provide a more individualized assessment of risk. This approach allowed us to utilize the variables along the entire range of values instead of using single cut points. Six hundred and eighty-nine patients were included and Tables 4 and 5 present the score for each risk factor and the predicted risk according to individual risk scores. Patients with total risk score between 0 and 4 had a 2-year progression risk of 3.8% (reference group). The risk was 26% (HR: 7.56, 95% CI: 3.77–15.2) for patients with a total score between 5 and 8, and for those with a score between 9 and 12, the risk of progression at 2 years was 51% (HR: 17.3, 95% CI: 8.63–34.8). When the score was >12, the 2-year progression risk was 73% (HR: 31.9, 95% CI: 15.4–66.3) (Fig. 4).

**Table 4:** Logistic regression equation to develop the risk score predicting progression risk at 2 years.

Risk factor	Coefficient	Odds ratio (95% CI)	*p* value	Score
FLC IU				
0–10 (reference)	–	–	–	0
>10–25	0.69	1.99 (1.15, 3.45)	0.014	2
>25–40	0.96	2.61 (1.36, 4.99)	0.004	3
>40	1.56	4.73 (2.88, 7.77)	<0.0001	5
M-protein				
0–1.5 (reference)	–	–	–	0
>1.5–3	0.95	2.59 (1.56, 4.31)	0.0002	3
>3	1.3	3.65 (2.02, 6.61)	<0.0001	4
BMPC				
0–15 (reference)	–	–	–	0
>15–20	0.57	1.77 (1.03, 3.06)	0.04	2
>20–30	1.01	2.74 (1.6, 4.68)	0.0002	3
>30–40	1.57	4.82 (2.5, 9.28)	<0.0001	5
>40	2	7.42 (3.23, 17.02)	<0.0001	6
FISH abnormality	0.83	2.28 (1.53, 3.42)	<0.0001	2

*FLC IU* involved to uninvolved serum-free light chain ratio.

**Table 5:** Predictive values of risk score tool.

Total risk score	Predicted risk at 2 years based on		Actual (% with 2-year progression)	Predictive value	
	Risk score	Full regression model		Positive	Negative
0	3.2	3.3	1 (1.3%)	25.8	n/a
2	6.2	6.1	3 (5.4%)	29.1	98.8
3	8.5	8.3	2 (2.6%)	31.5	97.1
4	11.6	11.1	3 (10.3%)	36.1	97.2
5	15.7	14.8	19 (19.2%)	37.7	96.3
6	20.8	19.4	11 (23.4%)	43	91.8
7	27	25	16 (27.6%)	46	89.9
8	34.3	31.5	21 (35%)	50.4	87.6
9	42.5	39	17 (48.6%)	55.4	85
10	51	46.9	18 (41.9%)	57	82.8
11	59.5	55	17 (50%)	63.2	81
12	67.5	62.9	13 (61.9%)	69.4	79.3
13	74.6	70.1	8 (50%)	72.5	77.9
14	80.5	76.5	11 (78.6%)	82.9	77.2
15	85.4	81.8	10 (83.3%)	85.7	76
16+	89.2	86.2	8 (88.9%)	88.9	75

## Discussion

The treatment paradigms for myeloma has evolved rapidly in the past decade with introduction of new therapies, leading to improved survival^18,19^. The mostly incurable nature of MM and a better understanding of the disease biology coupled with the prolonged precursor phase has inevitably directed our attention to developing strategies for prevention or early intervention with the goal of delaying onset of active disease or potentially eradicating the clone^20,21^. The redefinition of active myeloma was a first step in early intervention, with treatment initiation not solely based on CRAB criteria. The current study fills an important gap in terms of a risk stratification approach for the newly defined SMM that will allow us to design future clinical trials. We present here a simple risk stratification model that can be applied across the globe using easily available data to identify a subgroup of SMM patients with 50% progression risk at 2 years, defined by the presence of two or three factors among M-protein >2 g/dL, BMPCs infiltration >20%, or the ratio of involved to uninvolved sFLC >20. In this study, we used 2-year progression as the end point in order to maintain the approach used in the previous models but now excluding the ultrahigh risk patients with ≥80% risk of progression at 2 years who are essentially active M by current definition. This group represents a relevant population for early intervention and inclusion in trials in order to either prevent the MM development or potentially cure the disease.

The updated IMWG definition segregated from the classical SMM definition those patients with an imminent risk of progression to MM; now considered as MM. This subset of patients could potentially overestimate the progression risk in the classical SMM series and should therefore be excluded from the SMM risk stratification models. The Mayo Clinic group recently examined their cohort of SMM patients by removing those with MM according to the 2014 IMWG criteria, and they identified SMM at 50% risk of progression at 2 years based on M-protein (>2 g/dL), BMPC infiltration (>20%), and the ratio of involved to uninvolved sFLC (>20)^22^. Importantly, this international database of nearly 2000 SMM patients confirmed the Mayo Clinic 2/20/20 model, providing robust validation for this new categorization of SMM patients. In addition, the model is based on three features easily available at most centers. Moreover, a subanalysis conducted in a subset of patients with FISH information showed that the presence of any one of the cytogenetic abnormalities such as t(4;14), t(14;16), +1q, or del13q/monosomy 13 was an additional predictor of progression in this study. This is in line with a previous observation from the Mayo Clinic study that identified the presence of t(4;14), del17p), and/or hyperdiploidy as predictors of progression^10^. Altogether these data reinforce the concept that the genomic information such as mutations and translocation affecting MYC may contribute to refine the model, but this will require prospective validation in other studies^23^.

Different investigators have defined alternative models to evaluate the risk of progression from SMM to MM. Most have been based on measurements of tumor bulk: size of M-spike, sFLCs, type of immunoglobulin IgA versus IgG, proportion of clonal plasma cells >95% of aberrant cells, circulating plasma cells, or immune paresis. These models are useful and have driven the clinical research so far, being validated in clinical trials. However, new models have emerged based on relatively small series of patients utilizing new imaging assessments like PET-CT, genetic signatures, Bence Jones proteinuria, or dynamic models such as the evolution of the M-component and the decrease of hemoglobin, among others. Although many of them identify SMM patients with ≥50% risk of progression to MM within the first 2 years since diagnosis, in clinical practice, physicians are frequently confused about what model to use to define the risk of progression in SMM; moreover, many ongoing clinical trials in SMM use different inclusion criteria, which may be a confounding factor upon analyzing the efficacy of new drugs/combinations in this setting. Taken together, this model derived from an international SMM population with commonly available and reproducible biomarkers could be employed as a standard in registration trials as well as routine clinical practice.

The phase 3 trial conducted by the ECOG group evaluating single-agent lenalidomide versus observation in SMM patients showed a significant benefit in progression-free survival for the high-risk subset defined as in the current study using the 2/20/20 model^7^. Moreover, the same group decided to amend their new phase 3 trial comparing Ld versus Ld plus daratumumab in high-risk SMM in order to introduce the 2/20/20 model as inclusion criteria. The ASCENT trial conducted by the International Myeloma Foundation with a curative strategy is using the same model.

While we used 2-year progression as the end point to define a high-risk SMM population, we have also created a more precise and individualized scoring tool to classify individuals by risk of progression using the entire spectrum of values for each patient (in place of dichotomous division) including M-spike, BMPC infiltration, and sFLC ratio. Accordingly, this scoring tool is able to precisely identify SMM patients with extremely low risk of progression at 2 years (close to MGUS), as well as SMM with a risk of progression at 2 years even >50%. Thus, using this risk scoring, SMM patients with total risk score of 1 have 90% of probability of not developing MM in 2 years (negative predictive value (NPV) = 90%), while for those patients with total risk score of 9, the probability of developing MM in 2 years will be of 93% (positive predictive value = 93%). However, the identification of SMM patients who will not progress with near certainty (100% NPV) is difficult, and in the subgroup of patients with total risk score of 0, the risk does exist.

There are some limitations in this study because of its retrospective nature as well as the missing data observed for some variables that may have led to their exclusion in the multistep process. This is the case for the presence of immune paresis, percentage of plasma cells with aberrant phenotype or circulating plasma cells, or the evolution of the M-component and the decrease in hemoglobin. In addition, differences in the specific methodology used for FISH may vary from institution to institution. One additional limitation is its complete reliance on clinical features. It has been recently shown that the mutational landscape, particularly mutations in the RAS family as well as c-Myc alterations, may independently predict progression risk^24^. Moreover, the transition process from SMM to MM could also involve growth of preexisting clones due to a more permissive bone marrow microenvironment^25,26^. The current study also does not factor in other demographic factors such as race as the numbers were insufficient to explore this.

In summary, our study identifies a subgroup of SMM patients with 50% progression risk at 2 years from diagnosis based on the presence of two or three factors among M-protein (>2 g/dL), BMPC infiltration (>20%), or the ratio of involved versus uninvolved sFLC (>20). This model is easily reproducible and available worldwide, could be used to identify high-risk SMM patients in the context of clinical research, and will contribute in the near future to be able to offer early treatment to a more homogeneous subgroup of SMM patients. Availability of FISH data can improve the model and should be considered in all patients diagnosed with SMM. In addition, development of a scoring system allows for more individualized risk assessment. The trials going on and incorporating these features for the selection of the high-risk SMM patients will contribute to validate the model and ancillary biological studies can help to optimize it in the future. Future studies should focus on incorporating other tumor cell characteristics such as the presence of mutations as well as alterations in the tumor microenvironment, especially changes in the immune parameters.
